# Comparative Analysis of the Bioactive Compounds in Chicken Cartilage: Protective Effects of Chondroitin Sulfate and Type II Collagen Peptides Against Osteoarthritis Involve Gut Microbiota

**DOI:** 10.3389/fnut.2022.843360

**Published:** 2022-03-30

**Authors:** Hongru Zhang, Liwei Qi, Qingshan Shen, Ruiqi Wang, Yujie Guo, Chunhui Zhang, Aurore Richel

**Affiliations:** ^1^Key Laboratory of Agro-Products Processing, Ministry of Agriculture and Rural Affairs, Institute of Food Science and Technology, Chinese Academy of Agricultural Sciences, Beijing, China; ^2^Laboratory of Biomass and Green Technologies, University of Liege-Gembloux Agro-Bio Tech, Gembloux, Belgium

**Keywords:** chicken cartilage, chondroitin sulfate, collagen peptides, osteoarthritis, gut microbiota

## Abstract

This study was designed to explore osteoarthritis (OA) treatment from bioactive compounds of chicken cartilage food supplements. The OA rat model induced by sodium iodoacetate was used to evaluate the treatment effect *in vivo*. In this study, we used animal experiments to show that oral chondroitin sulfate (CS), cartilage powder, and type II collagen peptides could increase the athletic ability of rats and reduce inflammatory cytokine levels in serum or synovial fluid, including prostaglandin E_2_, tumor necrosis factor-α, interleukin (IL) 1β, IL-6, and IL-17. CS displayed the best treatment effect against OA. The morphological structure of articular cartilage indicated that CS could significantly improve cartilage tissue morphology and reduce OA score. Oral CS slowed down the development of OA by modulating gut microbiota. These results provided a useful scientific basis for the high-value utilization of chicken cartilage.

## Introduction

Osteoarthritis (OA) is a common chronic joint disease that is present globally. It has progressive articular cartilage degradation, affecting an increasing number of people in the elderly population, including the age group of 50–70 years (1, 2). The pathogenesis of OA relates to complex interactions among multiple pathways, resulting in the loss of structural components of joints, inflammation, and senescence of chondrocytes. The accurate molecular pathological mechanisms of cartilage loss and degradation are still not absolutely understood. The pathophysiology seems to be related to multiple microtraumas to the joint cartilage and the formation of extracellular matrix fragments (EMFs). EMFs activate extracellular signal-regulated kinase 1/2 (ERK1/2), p38 mitogen-activated protein kinases (p38MAPK), and c-Jun n-terminal kinase (JNK) by binding to the a5b1 integrin receptor, CD44, and TLRs on synovial chondrocytes and macrophages. Subsequent activation of protein-1 (AP-1) and nuclear factor κB (NF-κB) enhances the expression of matrix metalloproteinases (MMPs) for the cleavage of EMFs. Increased expression of MMPs is accompanied by increased synthesis of proinflammatory cytokines. Proinflammatory cytokines maintain chondrocyte activation and promote the formation of MMPs, aggrecanase, reactive oxygen intermediates, nitric oxide (NO), and lipid-derivative inflammatory mediators (prostaglandins and leukotrienes). These substances will both enhance the catabolic activity of chondrocytes and destroy the cartilage matrix.

In the clinic, it is very difficult to restore cartilage structure once it is damaged. The classical medication treatments of OA include acetaminophen, nonsteroidal anti-inflammatory drugs, and glucosamine sulfate for the abatement of pain and symptoms in the clinical setting. However, long-term oral nonsteroidal anti-inflammatory drugs can cause a series of side effects, including renal impairment and gastric ulceration. Thus, the natural bioactive ingredients derived from food have aroused increasing interest due to their sustainability and minimal side effects.

To discover safe, efficacious, and cost-effective bioactive compounds for managing OA symptoms, some food-derived ingredient supplements have been thought to exert beneficial effects on OA, such as type II collagen (CII), chondroitin sulfate (CS), *Spinacia oleracea* extract, hesperetin, *Boswellia* spp., *Curcuma* spp., chamomile, curcumin, quercetin, berberine, psoralen, avocado soybean unsaponifiables, fish oil, olive oil, omega-3-fatty acids, and anthocyanin (3–11). In particular, oral supplements composed of cartilage and soft tissue matrix components have clinical data supporting positive patient-reported functional improvement in OA.

Chondroitin sulfate, a naturally occurring sulphated heteropolysaccharide, is composed of alternative sequences of d-glucuronic acid and n-acetylgalactosamine joined by glycosidic linkages. It mainly has two isomeric forms, namely, CS-A and CS-C, based on the position of the sulfate group on n-acetylgalactosamine (12). CS belongs to the oral symptomatic slow-acting drugs (SYSADOA) for the treatment of OA in Europe and other countries (13). It achieves its therapeutic efficiency by activating various receptors widely distributed in tissues, including annexin 6, CD36/CD44, hyaluronic acid receptor, nogo receptors, and toll-like receptor-4 (TLR4) (14). By binding to CD44 and ICAM1, CS might promote the release of interleukin-1 (IL-1) receptor-associated kinase-M (IRAK-M), an inhibitor of IRAK, or the release of MKP-1, which dephosphorylates MAPK. CS also activates TLR4, which reduces phosphorylation of ERK1/2 and p38MAPK (15). The effects mentioned above could reduce the nuclear translocation of NF-κB to suppress inflammatory cytokines. Some studies also have demonstrated the ability of CII to slow down the development of OA due to the promotion of extracellular matrix synthesis and reduction of joint degeneration (10, 11, 16–18). One possible mechanism is that soft tissue matrix components act as prebiotics to modulate gut microbiota (17). An increasing number of studies have demonstrated that gut microbiota influences human metabolism, immune system, and inflammatory state. Dysbiosis of intestinal microbiota ecology could lead to bone-related diseases (19). Gut microbiota shifts induced by antibiotics, a germ-free environment, or high fat are important potential factors in joint damage and OA. DNA of the gut microbiota can be found in the synovium of OA and rheumatoid arthritis in human cartilage, which is significantly different between the OA and control groups. This indicates similar trafficking of gut microbiota (dead or alive bacteria) between the gut and epiphysial bone marrow (20, 21). The curative effects of probiotics (*L. casei, L. acidophilus*, and *Bifdobacterium longum*) on metabolic bone diseases have also been demonstrated (22, 23).

Although many studies have verified the anti-OA activity of cartilage-related dietary supplements, such as CII, glucosamine, and CS, the difference in therapeutic effect between type II collagen peptides (CP), CS, and cartilage powder (CPw) is not clear. To the best of our knowledge, the therapeutic effect of CS and its effects on the gut microbiota of OA have not been investigated *in vivo*. We have less knowledge about the relationship between CS from chicken cartilage and its anti-OA effect through altering the gut microbiota. Therefore, in this study, we investigated the anti-OA effect of CS and its underlying mechanism in OA rats and clarified the mechanism of CS on the alleviation of inflammation through regulation of the microbiota in the intestine. We prepared CPw, CP, and CS from chicken cartilage, and their basic chemical characteristics were investigated by Fourier-transform infrared (FT-IR) spectroscopy and high-performance liquid chromatography (HPLC). Their treatment against OA was demonstrated through an animal experiment by athletic ability analysis, inflammatory cytokine levels, histopathological observation, and gut microbiota composition.

## Materials and Methods

### Raw Materials and Chemicals

Chicken cartilage (white-feathered chicken, 42 days) was purchased from Protil Biotechnology Co. Ltd. (Hebi, Henan Province, China). The cartilage was stored at −20°C until use. Papain and trypsin were purchased from Sigma Aldrich (St. Louis, MO, USA). The standard unsaturated chondro/dermato disaccharides ΔDi0 S (ΔUA-[1 → 3]GalNAc), ΔDi4 S (ΔUA-[1 → 3]-GalNAc-4 S), ΔDi6 S (ΔUA-[1 → 3]GalNAc-6 S), ΔDi2,4 diS (ΔDi-dis B, ΔUA-2 S-[1 → 3]-GalNAc-4 S), ΔDi2,6 diS (ΔDi-dis D, ΔUA-2 S-[1 → 3]-GalNAc-6 S), ΔDi4,6 diS (ΔDidis, ΔUA-4 S-[1 → 3]-GalNAc-6 S), and ΔDi2,4,6 triS (ΔDi tris, ΔUA-2 S-[1 → 3]-GalNAc-4 S,6 S) were purchased from Iduron Corporation (Alderley City, UK). Other reagents were supplied by Sinopharm Chemical Reagent Co. Ltd. (China).

### Preparation of CPw, CP, and CS

Cartilage extracts (CP and CS) were prepared according to the procedure described by a previous study (24). In brief, dry chicken cartilage and distilled water were placed into a hot-pressure apparatus in a solid-liquid ratio of 2:5 (w/w). The hot-pressure processing conditions were 0.1 MPa and 90 min. Then, a certain amount of distilled water was added to the above solution. The excess solid was removed by filtering gauzes. Distilled water and trypsin were added to the filtrate to ensure a trypsin content of 0.13% and a filtrate content of 2%. Then, enzymatic hydrolysis was conducted for 2 h at 60°C. Papain (0.1%) was added, and the solution was incubated for 2 h at 60°C. Papain (0.1%) was added, and enzymatic hydrolysis was continued at 60°C for 2 h. After the above reaction, the solvent was boiled for 5 min to inactivate the enzyme. To obtain CS and CP, the enzymatic solution was filtered through membranes. In detail, the solution was first filtered through a 0.45 μm membrane by a suction filter. Subsequently, CS and CP were obtained from the filtrate by cycling using a peristaltic pump (WT600-2J, Shenzhen Saiyatech Instrument Equipment Co. Ltd., China) equipped with a 10 kDa membrane. Finally, the solutions mentioned above were dried at −38°C for 72 h by a freeze dryer (FD-1A-50, Boyikang Experimental Instrument Co. Ltd., China) and used for further experiments.

To remove excess moisture, chicken cartilage was dried for 48 h in an oven. Then, the dried cartilage was smashed to powder (1–100 μm) using a pulverizer. The CPw was stored in a dryer at 23 ± 2°C for follow-up experiments.

### Molecular Weight Distribution

The molecular weight of the CP sample was analyzed using an HPLC apparatus (Agilent 1260, Agilent Technologies, Walnut Creek, CA, USA) equipped with DAWN HELEOS-II (Wyatt Technology Corporation, America) and Optilabr EX (Wyatt Technology Corporation, USA) detectors coupled with a TSK gel G4000PWxl column (7.8 × 300 mm, TOSOH, Tokyo, Japan), following the previous study (25). The mobile phase included 0.1% trifluoroacetic acid, 45% acetonitrile, and 54.9% ultrapure water (ultrapure water machine, Milli-Q Synthesis, Millipore Corporation, USA). The standard was composed of Gly-Sar (146 Da), Gly-Gly-Tyr-Arg (451 Da), bacitracin (1,422 Da), aprotinin (6,511 Da), and cytochrome C (12,327 Da).

### Amino Acid Analysis

Total amino acid data were analyzed using an amino acid automatic analyser. In brief, samples were placed into hydrolysis tubes. Then, 6 M HCl was added to the tubes and thoroughly blended. After filling the samples with nitrogen for 1 min to purge air, the tubes were sealed and placed in a laboratory electric oven (PH-010, Shanghai Yiheng Scientific Instrument Co. Ltd., China) at 110°C for 24 h. Finally, the solution was filtered using a 0.45 μm filter membrane for amino acid detection by an amino acid automatic analyser (L-8900, Hitachi Ltd., Japan).

### Fourier-Transform Infrared Spectroscopy

The FT-IR spectra of CS standard and CS sample were detected using an FT-IR spectrometer (Tensor-27, Bruker Company, Germany). KBr was ground and was used as the background. The dried sample samples were mixed with KBr and then compressed into tablets. The FT-IR spectra were measured in the mid-IR region with a wave number ranging from 4,000 to 400 cm^−1^ and a spectral resolution of 4 cm^−1^.

### Enzymatic Treatment and Constitutive Disaccharide Determination

The CS sample was dissolved in ultrapure water at a concentration of 5 mg/ml, and 50 μl chondroitinase ABC enzyme (10 units/ml) was added to the CS solution for enzymatic hydrolysis at 37°C for 5 h. Then, the above solution was boiled at 100°C for 10 min to inactivate the chondroitinase ABC enzyme. The HPLC apparatus (Agilent 1260, Agilent Technologies, Walnut Creek, CA, USA) equipped with a 150 mm × 4.6 mm stainless-steel Spherisorb 5-SAX column (5 μm, trimethylammoniopropyl groups Si-(CH_2_)_3_-N^+^ (CH_3_)_3_ in the Cl^−^ form, Phase Separations Limited, Deeside Industrial Park, Deeside Clwyd, UK) was used to test the unsaturated disaccharides in the solution, and the ultraviolet absorption wavelength was 232 nm. The detailed separation procedures were as follows: 50 mM sodium chloride (pH = 4.0) for 5 min, followed by a linear gradient from 5 to 35 min of 50 mM to 1.0 M sodium chloride (pH = 4.0). All flow rates were 1.0 ml/min. The standard disaccharides were approved for qualitative and semiquantitative analysis.

### Animal Experiments

The Sprague Dawley (SD) rats (male, aged 7–8 weeks, bodyweight 220 ± 20 g) were purchased from Hunan SJA Laboratory Animal Co. Ltd. (Hunan, China, license: SCXK 2019-0004). After 7 days of acclimatization, the rats were housed in a controlled environment at 25 ± 2°C and 55 ± 5% of relative humidity. The OA model rats were induced with sodium iodoacetate and fed a normal diet for 2 weeks. After the OA model was successfully established, the OA rats were randomly divided into five groups (*n* = 6 per group): (1) the sham operation group (sham group): free diet, drinking water, and oral gavage of physiological saline; (2) the OA group: free diet, drinking water, and oral gavage of physiological saline; (3) the CS group: free diet, drinking water, and oral gavage of CS (100 mg/kg/day); (4) the CP group: free diet, drinking water, and oral gavage of CP (365 mg/kg/day); (5) the CPw group: free diet, drinking water, and oral gavage of CPw (500 mg/kg/day); and (6) the active control group (AC group): free diet, drinking water, and oral gavage of diacerein (8.0 mg/kg/day). All rats were fed with a standard diet (purchased from Mediscience Ltd, Jiangsu, China) *ad libitum*. The detailed process of the animal experiment is shown in **Figure 2A**. After the experiment, the SD rats were given deep anesthesia using the injection of 1% sodium pentobarbital (1.2 ml/100 g). The synovial fluids were extracted from the left hind leg knee joint of rats using an injector. Cartilage tissues were also obtained from the left hind leg knee joint of rats.

### Rotating Rod Test and Ramp Test

The rotating rod test and the ramp test were conducted during the nutritional intervention period at weeks 0 (before the intervention), 1, 2, 3, and 4. The data of the rotating rod time (RRT) were observed using a rotating rod instrument. In brief, rats were individually placed on a rotating rod (7.5 cm diameter) and trained through a rotating rod instrument. Before testing, rats were accustomed to 2 min in the rotating rod, and then the speed of the rod was slowly increased from 5 r/min to 15 r/min over 5 min. The persistence times of the rats were obtained, and each rat was tested 3 times (interval time: 2 h). The average value was recorded as the RRT.

The ramp experiment was carried out as follows: the rats were placed on a 25° inclined plane and trained for 1 min. Then, the ramp angle (RA) gradually increased until the rat was about to slip off. The rats persisted in a certain skew angle for 5 s. Each rat was measured 3 times, with an interval of 2 h, and the average RA was taken.

### Inflammatory Cytokine Detection

To determine the expression level of inflammatory cytokines, the samples of synovial fluid and serum were collected and detected using relevant ELISA kits (Elabscience, Wuhan, China) according to the manufacturer's instructions. The inflammatory cytokine in serum, such as serum IL-1β, was marked as “serum-related inflammatory cytokine.” The inflammatory cytokine in synovial fluid, such as SF IL-1β, was marked as “SF-related inflammatory cytokine.”

### Histopathological Observation of Cartilage Tissue

After the rats were sacrificed, the cartilage tissues were dissected and the unwanted parts were removed. Cartilage tissues were rinsed with 0.85% normal saline to remove blood and stain and were stored in 4% paraformaldehyde for 24 h. The samples were decalcified, dehydrated, and embedded in paraffin. The tissues were stained with haematoxylin and eosin (H&E). The cellularity and morphology of the specimens were examined using a microscope (Olympus Inc., Tokyo, Japan). The histological score (OA score) of cartilage tissues was obtained through double-blind observations, with Mankin's score given by three independent investigators.

### Sequencing of 16S rRNA of Gut Microbiota

In the classification and identification of bacteria and archaea, 16S rRNA sequences have been widely used. 16S rRNA gene sequences contain hypervariable regions that can provide species-specific signature sequences for the identification of bacteria. Advances in third-generation sequencing allow the simultaneous identification of thousands of 16S rRNA sequences within hours, enabling metagenomic studies, such as the gut microbiome (26). The microbial total genomic DNA was extracted based on a previous study (27). A NanoDrop spectrophotometer and 1% agarose gel electrophoresis were used to test the DNA concentration and quality. The V3-V4 region of the bacterial 16S rRNA gene was amplified by PCR using the forward primer 338F (5′-ACTCCTACGGGAGGCAGCAG-3′) and the reverse primer 806R (5′-GGACTACHVGGGTWTCTAAT-3′). The amplicon quality was purified using VAHTSTM DNA Clean Beads (N411-03, Vazyme Biotech Co., Ltd., Nanjing, China), and then the final amplicons were quantified and pooled for subsequent sequencing. When the average quality score was below 20, the sequences were cut-off. Subsequently, the paired-end reads were assembled using FLASH software. Sequences were de-noised as follows: the ambiguous, homologous sequences and less than 200 bp reads were discarded. Reads with 97% of bases above a quality value of Q20 were retained. Then the clean reads were obtained by removing the chimeric reads, which was conducted using UCHIME software (version 8.1). To obtain operational taxonomic units (OTUs), the clean reads were conducted for primer sequence removal and cluster. Based on the taxonomic database of Silva, the representative reads were annotated and blasted.

### Statistical Analysis

Statistical significance was determined using ANOVA along with Duncan's multiple comparisons. A *P*-value of < 0.05 was considered statistically significant. Statistical analysis was performed using R project 4.0 (R Core Team). The ACE estimator index, Chao 1 estimator, Shannon diversity index, principal component analysis (PCA), and so on were conducted to analyse the microbiota structure using R project 4.0. Spearman's correlation analysis and PERMANOVA tests were conducted using R project 4.0. The linear discriminant analysis effect size (LEfSe, Galaxy version 1.0) was conducted to identify the significant differences between groups (28).

## Results and Discussion

### Characterization of CS and CP

#### Molecular Weight Distribution and Amino Acid Composition of CP

**Figure 1:**
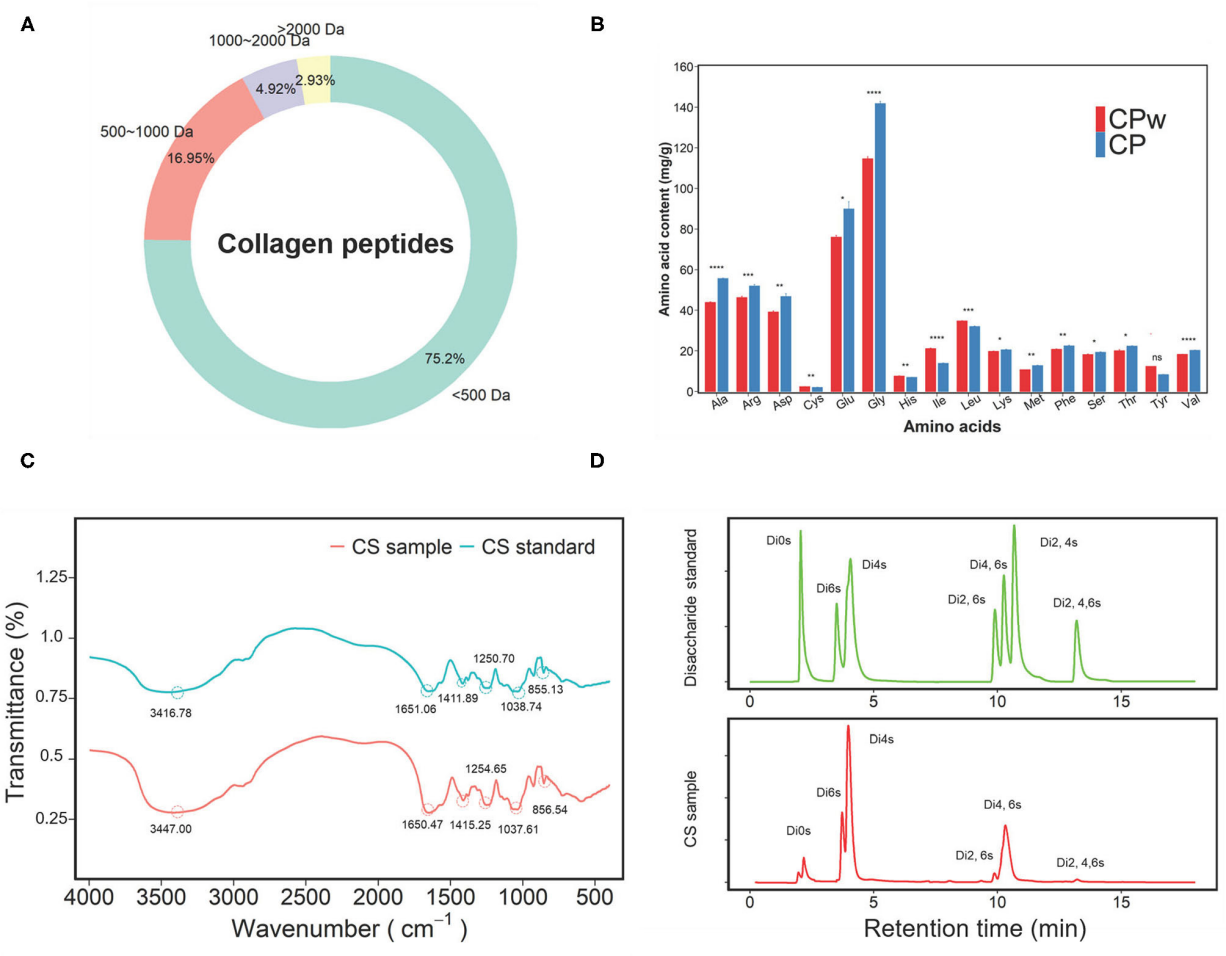
**(A)** The molecular mass distribution of CP. **(B)** The amino acid contents of CP and CPw. **(C)** The Fourier-transform infrared (FT-IR) spectra in the regions from 4,000 to 500 cm^−1^ of CS and CS standard. **(D)** The disaccharide chromatograms of CS sample and disaccharide standard. The symbols indicate statistical significance: ns: *P* > 0.05; *P ≤ 0.05, **P ≤ 0.01, ***P ≤ 0.001, ****P ≤ 0.0001.

To obtain more precise information on CS and CP samples, the molecular weight distribution and amino acid content were analyzed, respectively. As shown in Figure 1A, the molecular weight distribution of the CP sample was mainly 1,000 Da (92.15%), and the proportion of <500 Da was 75.2%. These results demonstrated that CP was mainly composed of small peptides. The molecular weight of collagen is about 300 kDa. It is generally accepted that small peptides are easily absorbed in the small intestine with high biological activity. The amino acid contents of CPw and CP samples are shown in Figure 1B. Except for Tyr, the remaining amino acid contents showed significant changes between CPw and CP samples. The contents of Ala, Arg, Asp, Glu, Gly, Lys, Met, Phe, Ser, Thr, and Val in CP were higher than those in CPw. The contents of Cys, His, Ile, and Leu in CP were lower than those in CPw. The proportions of amino acids were calculated to evaluate the amino acid composition of the sample. No obvious difference was observed in the amino acid proportion of total amino acid (%) between CPw and CP. Compared with CPw, the total amino acid content of CP increased, while the molecular weight distribution of CP decreased, with the amino acid proportion of CP unchanged.

#### Fourier-Transform Infrared Spectroscopy and Disaccharide Analysis of CS

The CS sample was qualitatively identified by FT-IR spectroscopy using the CS standard (CS-A) as the standard (Figure 1C). In the spectra, the region above 2,000 cm^−1^ was dominated by the OH stretching vibration. The bands at 1,651.06 and 1,650.47 cm^−1^ were due to the amide 1 band for the CS sample and CS standard, respectively. The bands at 1,250.70 cm^−1^ for the CS sample and at 1,254.65 cm^−1^ for the CS standard were assigned to S=O corresponding to the band assignment (29). The peaks at 852.2 cm^−1^ and 823.7 cm^−1^ are the characteristic peaks of chondroitin-4-sulfate (CS-A) and chondroitin-6-sulfate due to the C-O-S ring vibrations, respectively (30, 31). In this study, there was a distinct peak at 855.13 cm^−1^ in the CS sample, indicating that the main ingredient of the CS sample was CS-A. The bands at 1,038.74 cm^−1^ (CS sample) and 1,037.61 cm^−1^ (CS standard) were due to the C-O-C ring vibrations (32). The peaks of 1,651.06 cm^−1^ and 1,650.47 cm^−1^ were clearly observed in the CS sample and CS standard, indicating the presence of uronic acid.

In addition, to obtain accurate disaccharide composition of the CS sample, the unsaturated disaccharides of the CS sample were analyzed by HPLC (Figure 1D). Nonsulphated disaccharide ΔDi0 S (4.96%), ΔDi4 S (47.20%), and ΔDi6 S (12.78%) were found in the CS sample, and the content of 4-sulphated disaccharide in the CS sample was visibly higher than that of 6-sulphated disaccharide with a 4 S/6 S ratio of 3.58, which indicates that CS-A was the main ingredient of the CS sample. Interestingly, disulphated disaccharide and trisaccharide were found in the CS sample, including ΔDi2, 6S, ΔDi4, 6S, and ΔDi2, 4, 6S. In this study, the disaccharide composition of the CS sample was similar to the results of our previous study (24). These results indicate that CS was extracted successfully from chicken cartilage.

### Cartilage Extracts Promote Athletic Ability in OA Rats

**Figure 2:**
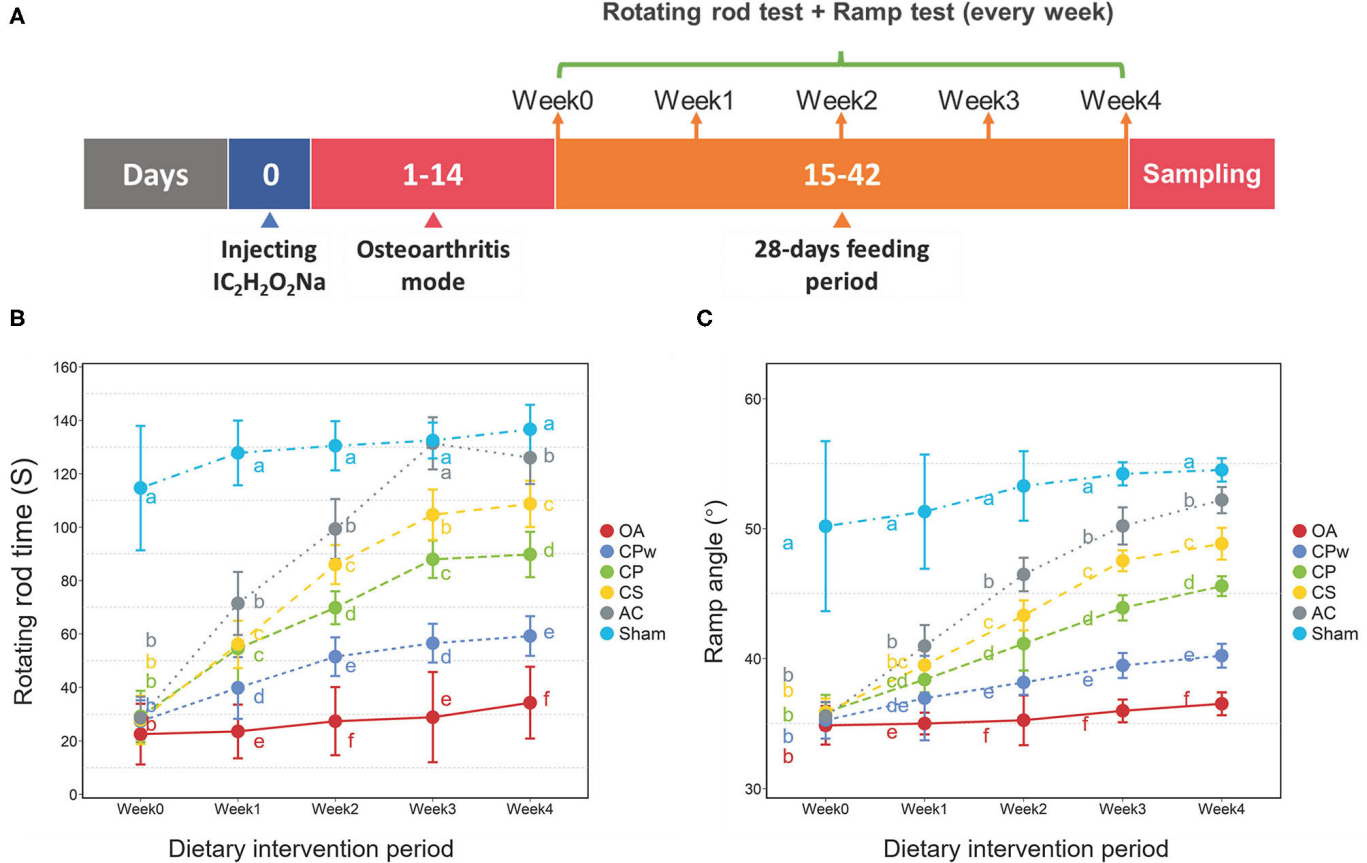
**(A)** Detail time nodes of the animal experiment. **(B)** Rotating rod time of different groups. **(C)** Ramp angle of different groups. Duncan's test was used to evaluate the significant differences between different groups. The different letters mean the significant difference between the two groups.

In clinical cases of OA, pain and swelling of joints are important clinical manifestations that lead to an exercise capacity decline in the individual. As shown in Figures 2B,C, the RRT and RA of rats were evaluated, respectively. There were slight variations in RRT and RA (*P* > 0.05) in the sham and OA groups from week 0 to week 4. However, the RRT of rats (CPw, CP, and CS groups) increased significantly with prolonged dietary intervention time. However, different groups showed diverse increase rates during weeks 0–4, and the CS group showed the highest increase rate, followed by the CP and CPw groups. In week 4, the RRT level of the CPw, CP, and CS groups trended to that of the sham group with different degrees (CS > CP > CPw). As shown in Figure 2C, the RA of different groups showed the same increasing trend as the RRT during weeks 0–4. Among the CS, CP, and CPw groups, the CS group also showed the highest increase rate, and the CPw group showed the minimum increase rate. In summary, the RRT and RA data showed a similar increasing trend in the cartilage extract groups (CPw, CP, and CS groups) during the dietary intervention. These results indicated that CPw, CP, and CS enhanced rat athletic ability. However, there were significantly different treatment effects on OA among cartilage extract groups. The CS group had the best therapeutic effect among the CS, CP, and CPw groups, while the CPw group had the weakest treatment effect. This might be caused by the macromolecular substances (collagen and polysaccharide) in CPw, which are difficult for the organism to digest, absorb, and utilize. Recent studies have shown similar results that oral CII could improve physical activities to alleviate OA (33). *In vivo* animal studies have demonstrated that CII acts through specific regulatory T cells in the gut for arthritis treatment. After stimulation, T cells migrate and concentrate in areas of inflammation, where they modulate local immunity by the antigen-specific immune response (34). Puigdellivol et al. showed that treatment with the dietary supplement containing hydrolysed collagen, CS, and glucosamine significantly reduced pain and improved locomotor function in patients with OA of the knee and/or hip (35). OA-induced subchondral bone changes and synovitis may be responsible for noxious irritation, while peripheral neuronal sensitization is an important feature that can lead to pain in normal activities such as walking (36). MMPs are known to degrade the cartilage and may play a role in pain-related vascular and nerve ingrowth. Mapp et al. showed that MMP inhibitor reduces joint damage, pain response, and osteochondral angiogenesis in OA rats (37). CS could decrease the synthesis of MMPs. By increasing anti-inflammatory molecules and reducing oxidative stress, CS showed antinociception and neuroprotection in joint damage-induced neuropathic pain in rats (38). Therefore, CS improved the athletic ability of OA rats in this study.

### Cartilage Extracts Improve the Morphology of Cartilage Tissue

**Figure 3:**
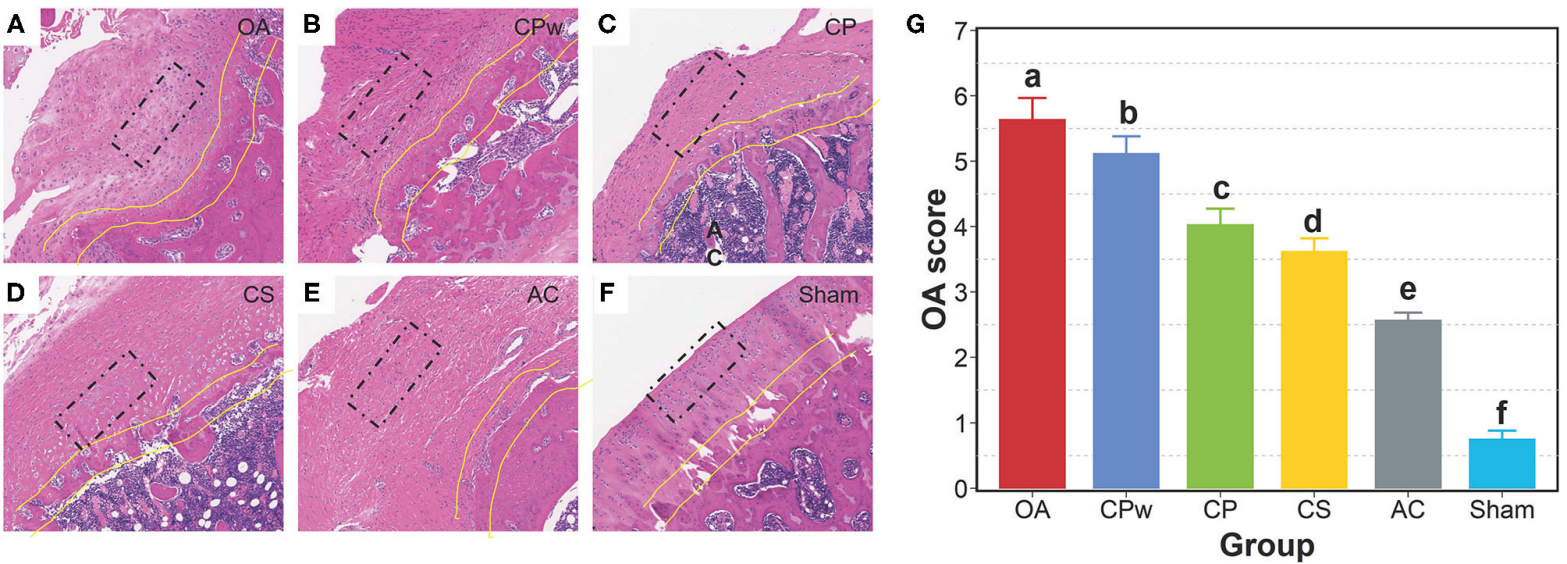
Morphological observation of cartilage tissue. **(A–F)** The H&E staining images of the OA, CPw, CP, CS, AC, and sham groups, respectively. **(G)** The OA score by the Mankin's score. The cell morphology is inside the black rectangle. The tide line is between the two yellow lines. The different letters mean the significant difference between the two groups.

As shown in Figures 3A–F, H&E-stained sections showed significant differences in morphological structure among groups. In the sham group, the chondrocytes of cartilage tissue were neatly arranged without clustering, and the tide line was clear. In contrast, H&E staining in the OA group showed destruction of tissue integrity, irregular arrangement of cells, and discontinuous tides. In the CPw, CP, and CS groups, the clustering of chondrocytes was markedly reversed. In the CS group, the surface of cartilage was intact, the cell arrangement was regular, and the space between the tissue and the tide line was intact. The mentioned H&E-stained images were also used to evaluate the OA score by Mankin's score method. As shown in Figure 3G, the OA score of the cartilage extract groups (CPw, CP, and CS groups) was significantly lower than that of the OA group. The OA score results agreed with the results of the H&E-stained images. The OA group showed the highest OA score, which indicates that the model of OA was established successfully. The OA score of the CS group was lower than that of the CPw and CP groups. Ren et al. showed a similar result that shark CS improved the cartilage tissue structure in an OA rat model (39). In OA rats, MMP-1 degrades ECM and activates other MMPs to further accelerate cartilage destruction. Inflammatory cytokines such as IL-1β and tumor necrosis factor-α (TNF-α) play an important role in the development of OA. IL-1β can upregulate the expression of NO, cyclooxygenase-2 (COX-2), prostaglandin E_2_ (PGE_2_), and MMPs to accelerate the degradation of cartilage. CS inhibits activation and nuclear translocation of NF-κB, which mediates the expression of MMPs, IL-1β, and TNF-α to reduce joint damage.

### Cartilage Extracts Modulate Inflammatory Cytokines in OA Rats

Inflammatory cytokines contribute to joint damage. IL-1 plays a role in the early stage of OA and affects the normal physiological function of chondrocytes. In addition to IL-1, a series of inflammatory cytokines in the interleukin family, such as IL-17 and IL-18, also play obvious roles in joint destruction. TNF-α initiates intracellular signal transduction by binding to the corresponding receptors of chondrocytes, thereby regulating their biological functions. It ultimately activates the most important NF-κB/p65 transcriptional pathways, which in turn secrete IL-6 and IL-8, leading to the aggravation of OA (40, 41). PGE_2_ is spontaneously secreted by the cartilage of OA and overexpressed in the synovial fluid of the OA joint. It promotes local vasodilation and activation and migration of neutrophils, macrophages, and mast cells, and it can activate nociceptors through prostaglandin E (EP) receptors (42). IL-10 is widely known as an anti-inflammatory cytokine produced by a variety of cell types. It can antagonize the expression of other cytokines, such as TNF-α, IL-1β, and IL-8, thereby controlling inflammation.

**Figure 4:**
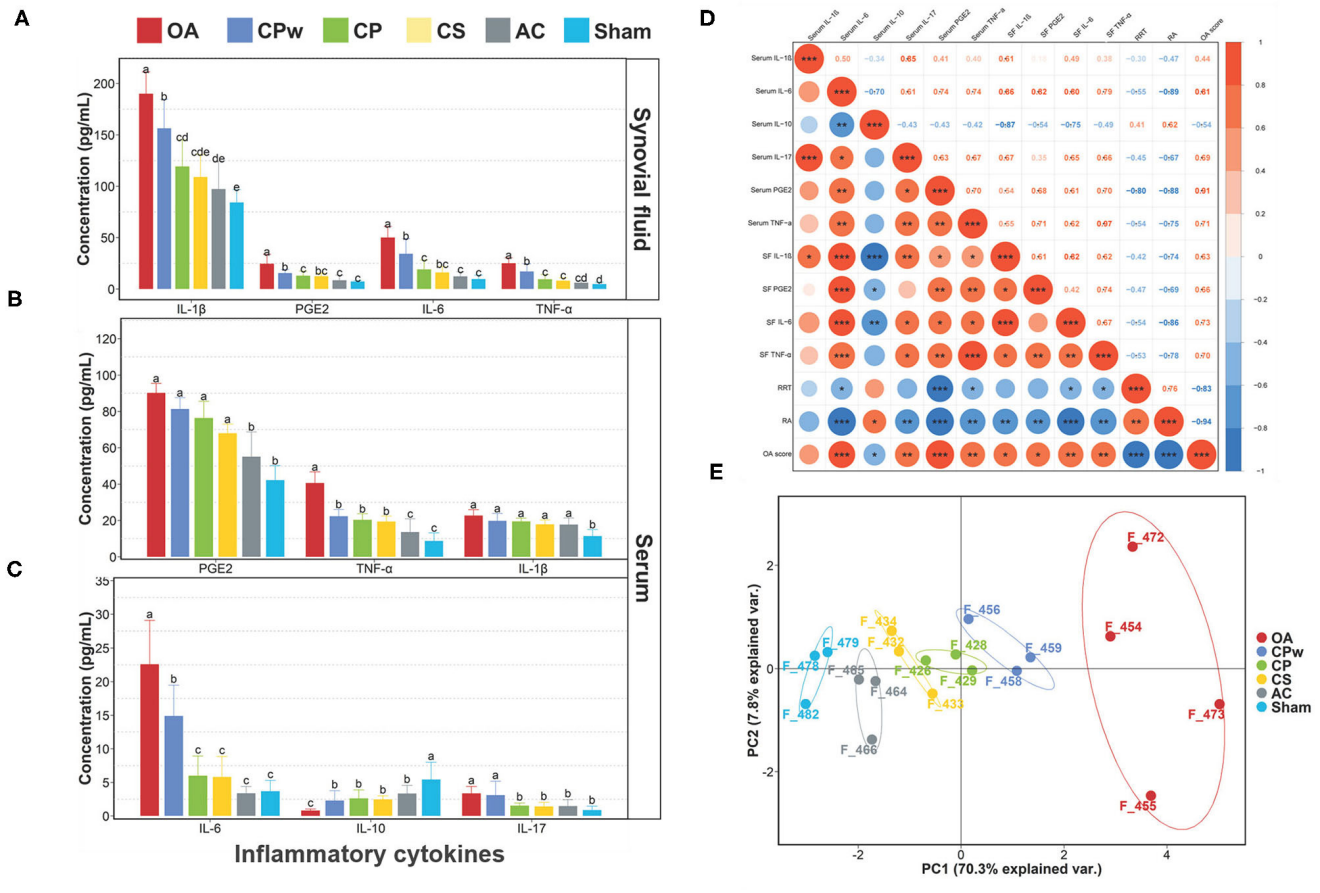
**(A)** IL-1β, PGE_2_, IL-6, and TNF-α levels of synovial fluid. **(B)** PGE_2_, TNF-α, and IL-1β levels of serum. **(C)** IL-6, IL-10, and IL-17 levels of serum. **(D)** Spearman's r correlations between the inflammatory cytokines and athletic ability. **(E)** Principal component analysis for different groups. Data were represented as mean ± SD. Duncan's test was used to evaluate the significant differences between different groups. The different letters mean the significant difference between the two groups. Significant correlations were noted by *P ≤ 0.05, **P ≤ 0.01 and ***P ≤ 0.001.

To evaluate the osteoarthritic treatment of CPw, CP, and CS at the molecular level, the inflammatory cytokine levels in synovial fluid were detected using ELISA kits. As shown in Figure 4A, compared with the OA group, the IL-1β, IL-6, TNF-α, and PGE_2_ levels of the cartilage extract groups (CPw, CP, and CS groups) showed significant declining phenomenon with different degrees (*P* < 0.05). However, all the inflammatory cytokine contents of the cartilage extract groups were higher than those of the AC and sham groups. Interestingly, the CS group showed the lowest inflammatory cytokine levels (IL-1β, PEG_2_, IL-6, and TNF-α) among the cartilage extract groups, and the CPw group showed the highest levels. The CPw group showed a weaker treatment effect than the CS and CP groups due to the stable physical and chemical structure of CPw, inhibiting its digestion and hydrolysis in the gastrointestinal tract. Crowley et al. demonstrated that non-denatured CII-treated subjects showed significant enhancement in daily activities at the end of 90-day treatment (11).

To further study the effect of cartilage extracts on inflammatory cytokines in metabolism, we also collected serum samples for follow-up detection. As shown in Figures 4B,C, among different groups, the content of inflammatory cytokines in serum showed a similar phenomenon compared with that in synovial fluid. For TNF-α, IL-10, and IL-6, there were significant differences between the OA group and the other groups. However, for TNF-α and IL-10, the CPw, CP, and CS groups did not show a significant difference (*P* > 0.05). The IL-10 content of the CP and CS groups was lower than that of the CPw group. In PEG_2_ and IL-1β, the cartilage extract groups did not show a significant difference (*P* > 0.05) compared with the OA group. Based on the above results, CP, CS, and CPw could decrease the level of inflammatory cytokines to achieve OA treatment. Among them, CS had the best treatment effect in the regulation of inflammatory cytokines.

To study the correlation of OA-related indicators, Spearman's correlation analysis was performed (Figure 4D). Both RRT and RA showed positive correlations with serum IL-10, and the correlation between RA and serum IL-10 was significant (*P* < 0.05). RA was significantly negatively correlated (*P* < 0.05) with most inflammatory cytokines except serum IL-1β. RRT was significantly negatively correlated (*P* < 0.05) with serum IL-6, serum PEG_2_, serum TNF-α, SF IL-6, and SF TNF-α. There were positive correlations between inflammatory cytokines. Serum IL-10 showed negative correlations with other inflammatory cytokines. This is consistent with the previous statement that IL-10 inhibits the expression of inflammatory cytokines (TNF-α, IL-1β, IL-8, etc.). IL-10 is responsible for inhibiting the expression of family of metalloproteinases (MMPs). The main reason may be that IL-10 stimulates the synthesis of IL-1β antagonists, which are IL-1Ra and the tissue inhibitor of metalloproteinases-1 (TIMP-1) (43). Serum IL-10 showed a positive correlation (*P* < 0.05) with RA and a negative correlation (*P* < 0.05) with the OA score. It has been verified that IL-10 contributes in stimulating the synthesis of CII and aggrecan, which are the main ingredients of EMFs.

The data of athletic ability, the morphology of cartilage, and inflammatory cytokines were used to indicate the difference between different experimental groups (Figure 4E) *via* PCA. The PCA plot between groups showed a sum explanation of 76.9% (PC1: 68.7%, PC2: 8.2%). The samples of the OA group could be clearly distinguished from the samples of the other groups. The samples from the CS, CP, and CPw groups were close to those of the sham group in distance. These results indicated that CS, CP, and CPw alleviated the changes in indicators caused by OA.

### Cartilage Extracts Modulate the Gut Microbiota in OA Rats

Environmental factors, including diet, lifestyle, antibiotic treatment, and probiotics, affect the alteration of the gut microbiota composition. Gut microbiota, such as *Lactobacillus*, directly or indirectly interact with immune and epithelial cells by modulating inflammatory cytokines and oxidative stress (44). The gut microbiota indirectly acts on the central nervous system and immune system through the production of metabolites such as short-chain fatty acids (SCFAs), metabolites of tryptophan, and bile acids. Decreased butyrate production is associated with the activation of neutrophils and macrophages (21). Hu et al. showed the modulation of the gut microbiota by CS from *Acaudina molpadioides* can improve chronic inflammation and increase the levels of fecal SCFAs (45). Thus, the effect of CS on modulating the gut microbiota was studied by 16S rRNA gene sequencing.

**Figure 5:**
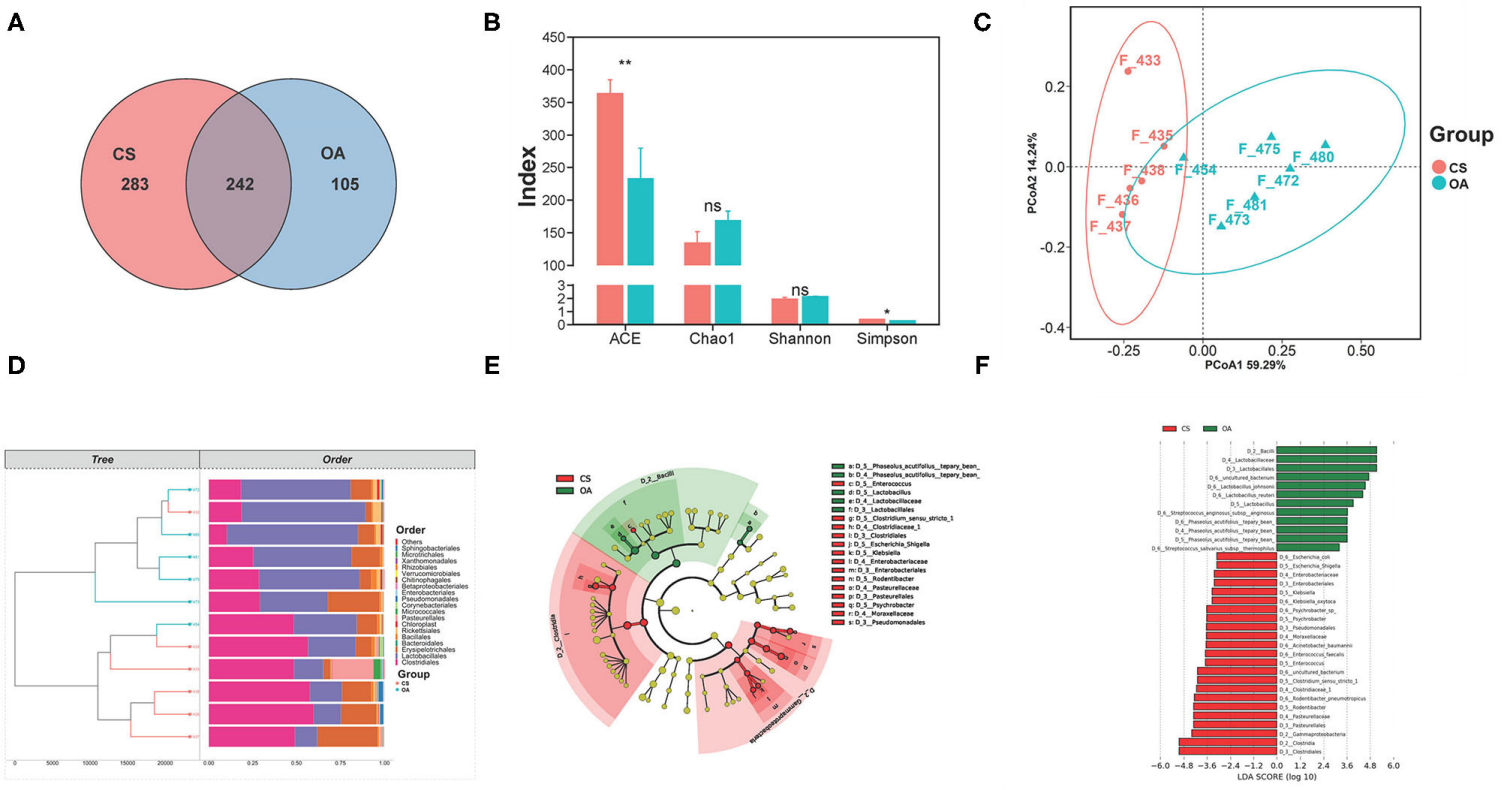
**(A)** Venn diagram of the OA and CS groups at OTUs level. **(B)** Effects of CS on the alpha diversity of the gut microbiota from the OA rats. **(C)** The PCA plot of samples from the OA and CS groups. **(D)** Clustering analysis (at order level) of the gut microbiota among samples from the OA and CS groups. **(E)** Linear discriminant analysis effect size. **(F)** Taxonomic cladogram derived from LEfSe with an LDA score >2.0. The symbols indicate statistical significance: ns (*P* > 0.05), *P ≤ 0.05, **P ≤ 0.01.

Based on the sequencing results, the total number of OTUs in the OA and CS groups was 623, and there were 105 specific OTUs in the OA group and 283 specific OTUs in the CS group (Figure 5A). Changes in gut microbial diversity (expressed by Shannon and Simpson) and richness (expressed by Chao 1 and Abundance-based Coverage Estimator, ACE) are shown in Figure 5B. The species diversities within microbiome samples (α diversity) were evaluated using the Shannon index, without significant differences in the CS group (*P* > 0.05, CS group vs. OA group). The same results were achieved in the Chao1 index. There were significant differences in the ACE index and the Simpson index between the OA and CS groups (P < 0.05). As shown in Figure 5C, the hierarchical clustering analysis at the order level illustrated distinct bacterial clusters that responded to the CS and OA groups, and only one sample from the CS group was incorrectly clustered to the OA group. The above results revealed a significant difference in gut microbiota structure between the CS and OA groups. The diversities between microbiome samples (β diversity), as determined by the principal components analysis (PCA), showed two clusters that were relatively separated, suggesting the distinct bacterial differences between the two groups (Figure 5D), and the main two PCA components accounted for 59.29 and 14.24%, respectively. The PERMANOVA test showed the remarkable effect of CS on modulating bacterial structures between groups (bray curtis, 999 mutations, F = 7.84, R2 = 0.53, *P* = 0.024). Furthermore, LEfSe was conducted to identify the biomarkers characterizing microbial differences between the CS and OA groups (Figure 5F). Cladograms are shown in Figure 5E. In OA rats, *Lactobacillus johnsonii* and *L. reuteri* were the dominant bacteria. A similar study reported that anaerobes such as *L. salivarius* and *Atopobium* spp. were enriched in salivary and dental samples from subjects with rheumatoid arthritis (46). Thus, to further study the gut microbiota modulation of CS, the relative abundance of *Lactobacillus* at the genus level was analyzed. It decreased significantly in the CS group, compared with that in the OA group. The increased abundance of the phylum *Proteobacteria* has been demonstrated to be a marker for an unstable microbial community and a risk factor for human disease (47, 48). In particular, oral supplementation was associated with a reduction in *Proteobacteria* and an increase in *Bacteroidetes* that inhibit stress-induced intestinal inflammation. In this study, we found a difference in the abundance of the phylum Proteobacteria, between OA group and CS group, but the difference did not reach a statistically significant level. The CS group indicate an increase in the abundance of Bacteroidetes, compared to the OA group (Supplementary Figure 1). These results indicate that CS can modulate the gut microbiota.

**Figure 6:**
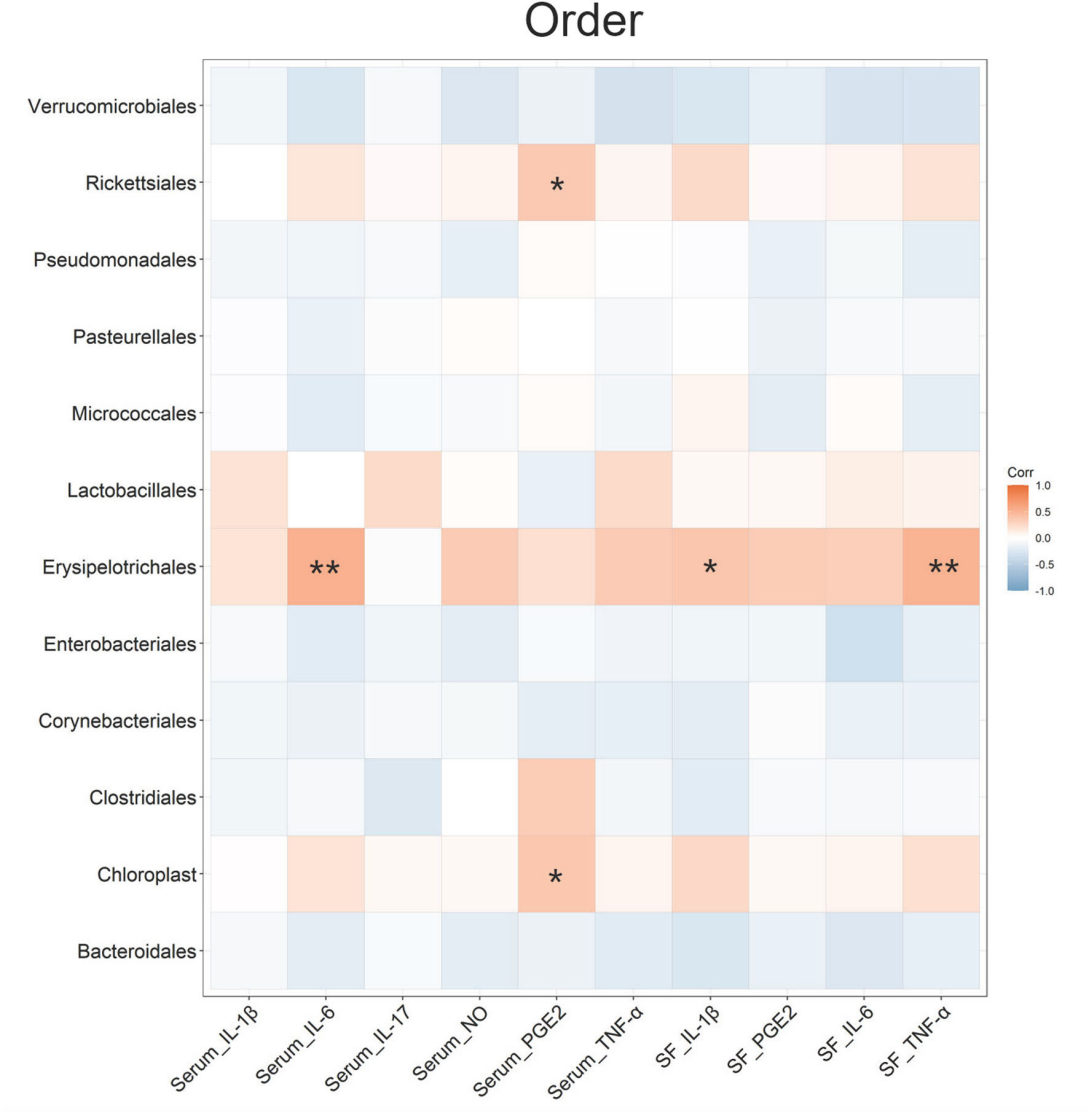
Spearman's r correlations between the inflammatory cytokines and the gut microbiota in terms of order level. Significant correlations were noted by *P ≤ 0.05, **P ≤ 0.01.

A Spearman's correlation analysis was conducted to estimate the association between the OA-related cytokines and gut microbiota at the order level (Figure 6). It indicated that 3 kinds of bacteria (*Rickettsiales, Chloroplast*, and *Erysipelotrichales*) at the order level had significant correlations with serum inflammatory cytokines or SF inflammatory cytokines. In particular, *Erysipelotrichales* showed a positive correlation with serum IL-6, SF IL-1β, and SF TNF-α. The relative abundance of *Erysipelotrichales* decreased in the CS group compared with that in the OA group (P < 0.05). A similar study demonstrated that collagen-induced arthritis rats had a higher abundance of *Erysipelotrichales* than control rats (49). The Spearman's correlation analysis was also indicated at the family, genus, and species levels (Supplementary Figure 2). *Lactobacillaceae* (family level) and *Lactobacillus* (genus level) showed significant correlations with SF IL-6. This was consistent with the result of LEfSe that *Lactobacillus* was the biomarker of OA. Xie et al. found that the genus *Lactobacillus* abundance and family *Lactobacillaceae* abundance of OA people were significantly higher than those of healthy people (50). Liu et al. indicated that the fecal microbiota of patients with rheumatoid arthritis showed significantly more *Lactobacillus* (10.62 ± 1.72 copies/g) than that of the control group (8.93 ± 1.60 copies/g) (51). These results indicated a potential relationship between *Lactobacillus* communities and OA. This needs to be further confirmed in follow-up studies.

## Conclusion

This study found that cartilage extracts (CPw, CP, and CS) exhibited treatment effects on sodium iodoacetate-induced OA rats, and CS displayed the best anti-OA activity. CS significantly enhanced the athletic ability of OA rats. Additionally, CS significantly downregulated the related inflammatory cytokine levels (IL-1β, IL-6, PGE_2_, TNF-α, etc.) in serum or synovial fluid. The H&E staining results indicated that CS could reverse cartilage tissue morphology, and the CS group showed the minimum Mankin's score. Additionally, CS modulated the gut microbiota in the small intestine, which is essential for intestinal metabolite production. This study demonstrated that the therapeutic effect of CS for OA was better than that of CP and CPw. These findings provide a scientific basis for the high-value utilization of livestock coproducts.

## Data Availability Statement

The datasets presented in this study can be found in online repositories. The names of the repository/repositories and accession number(s) can be found below: NCBI SRA; PRJNA810508.

## Ethics Statement

The animal study was reviewed and approved by Institute of Food Science and Technology, Chinese Academy of Agricultural Sciences Animal Ethical and Welfare Committee.

## Author Contributions

HZ: data curation, formal analysis, methodology, and writing - original draft. LQ and HZ: visualization. QS, RW, HZ, and YG: investigation. AR, CZ, QS, RW, YG, LQ, and HZ: writing - review and editing. CZ: conceptualization, resources, project administration, and funding acquisition. All authors contributed to the article and approved the submitted version.

## Funding

This work was supported by the National Natural Science Foundation of China (32072156); National Agricultural Science and Technology Innovation Project (CAAS-ASTIP-2020-IFST-05); National Key Research and Development Plan of China (2021YFD2100804).

## Conflict of Interest

The authors declare that the research was conducted in the absence of any commercial or financial relationships that could be construed as a potential conflict of interest.

## Publisher's Note

All claims expressed in this article are solely those of the authors and do not necessarily represent those of their affiliated organizations, or those of the publisher, the editors and the reviewers. Any product that may be evaluated in this article, or claim that may be made by its manufacturer, is not guaranteed or endorsed by the publisher.

## Supplementary Material

Supplementary Figure 1 **(A)** Relative abundance of *Proteobacteria*. **(B)** Relative abundance of *Bacteroidetes*. The symbols indicate statistical significance: ns (*P* > 0.05).

Supplementary Figure 2 Spearman's r correlations between the inflammatory cytokines and the gut microbiota. **(A)** Family level. **(B)** Genus level. **(C)** Species level. Significant correlations were noted by ^*^*P* ≤ 0.05, ^**^*P* ≤ 0.01.
