# Supplementary figures and images for: Comparative Analysis of the Bioactive Compounds in Chicken Cartilage: Protective Effects of Chondroitin Sulfate and Type II Collagen Peptides Against Osteoarthritis Involve Gut Microbiota
Source: Front Nutr. 2022 Mar 30;9:843360. doi: 10.3389/fnut.2022.843360 (PMC9005812; doi:10.3389/fnut.2022.843360)

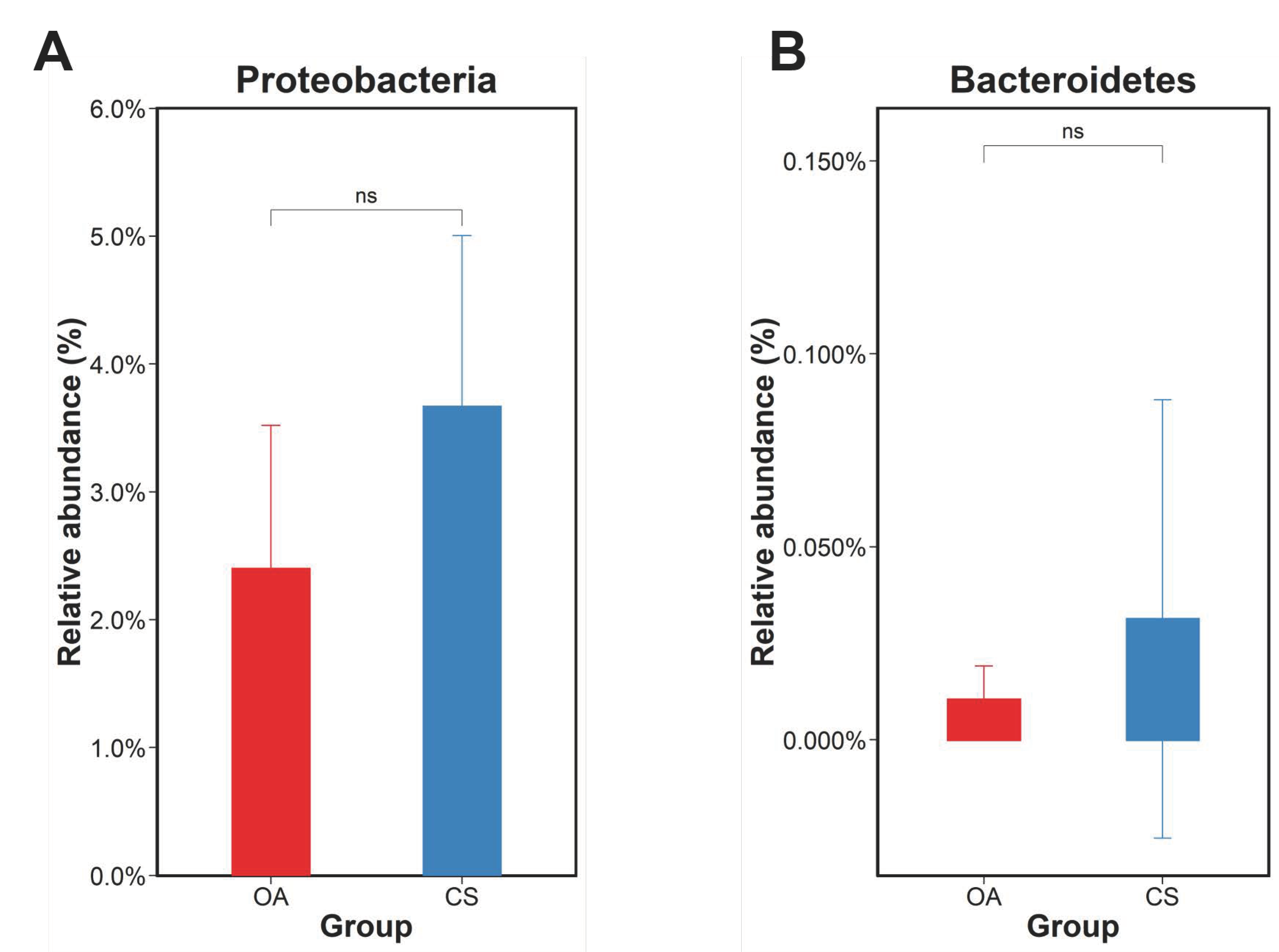

Supplement: Supplementary Figure 1 — (A) Relative abundance of Proteobacteria. (B) Relative abundance of Bacteroidetes. The symbols indicate statistical significance: ns (P > 0.05). [file Image_1.tif]

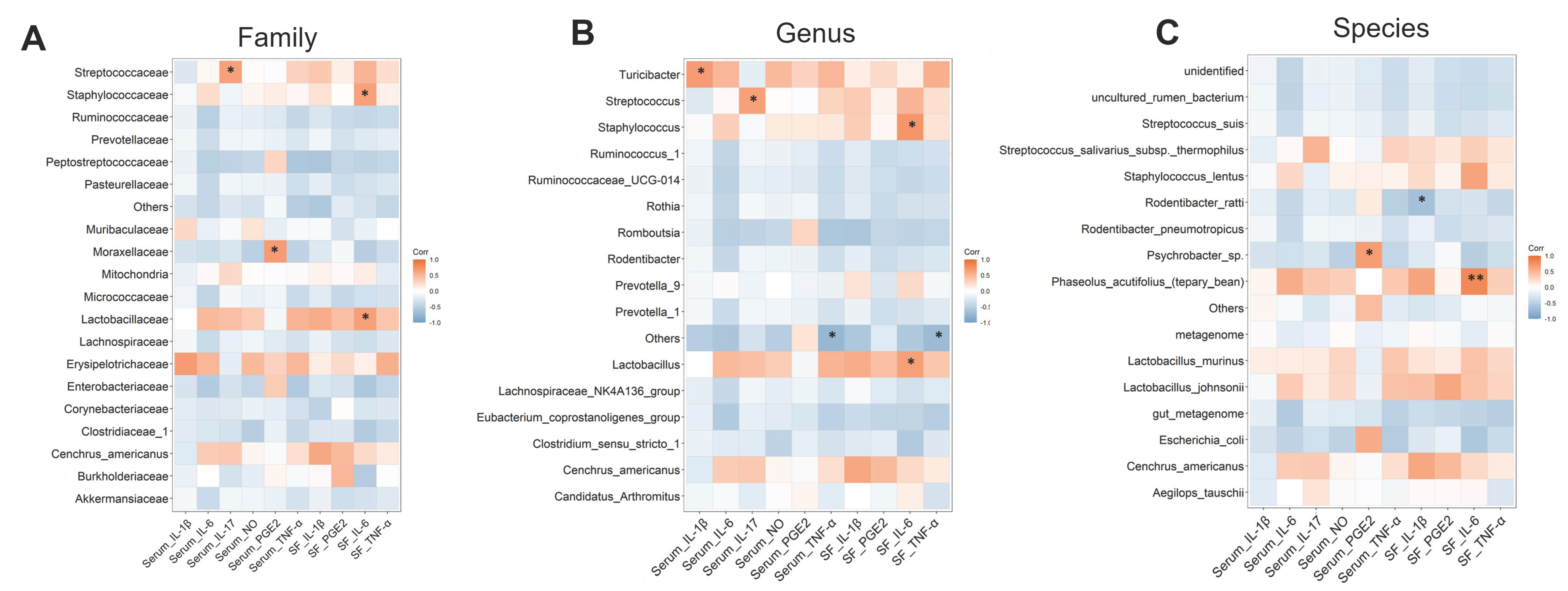

Supplement: Supplementary Figure 2 — Spearman's r correlations between the inflammatory cytokines and the gut microbiota. (A) Family level. (B) Genus level. (C) Species level. Significant correlations were noted by *P ≤ 0.05, **P ≤ 0.01. [file Image_2.TIF]
